# Clinical and epidemiological characteristics of multiple sclerosis in the southern region of the Republic of Azerbaijan

**DOI:** 10.12669/pjms.41.2.11373

**Published:** 2025-02

**Authors:** Rahim R. Aliyev, Shahla N. Mehtiyeva, Rana K. Shiraliyeva

**Affiliations:** 1 Rahim R. Aliyev, MD, PhD Associate Professor of Department of Neurology, Azerbaijan Medical University, Bakikhanov Street 23, Baku, Azerbaijan; 2 Shahla N. Mehtiyeva, MD, PhD Associate Professor of Department of Neurology, Azerbaijan Medical University, Bakikhanov Street 23, Baku, Azerbaijan; 3 Rana K. Shiraliyeva, MD Doctor of Medical Sciences, Full Professor of Neurology, Head of Department of Neurology and Clinical Neurophysiology, Azerbaijan State Advanced Training Institute Named After A. Aliyev, Tbilisi Ave. 3165, Baku, Azerbaijan

**Keywords:** Azerbaijan, Delayed Diagnosis, Epidemiology, Incidence, Multiple Sclerosis, Prevalence

## Abstract

**Objectives:**

To investigate the clinico-epidemiological characteristics of multiple sclerosis (MS) in the southern region of the Republic of Azerbaijan, which has a population of 930,601.

**Methods:**

This prospective, single-center, longitudinal study was conducted from January 1, 2013, to December 31, 2022. MS patients in this region underwent examination and treatment under a State Program. During this period, 123 individuals visited the Neurological Center, and 104 confirmed MS cases were included in a digital database. Age-standardized incidence and prevalence rates per 100,000 were calculated using world and European population standards. Data analysis was performed using IBM SPSS Statistics 27.

**Results:**

The mean diagnostic age was 36.31±6.93 years, higher than the countrywide average of 34.9 years. The average diagnostic delay was 7.01 years, significantly longer than the national average of 5.2 years. Regional MS prevalence was 10.96/100,000, with higher urban rates but no significant sex difference. The average 10-years incidence of MS was 0.94±0.32/100,000. Compared to neighboring regions, such as Iran’s East Azerbaijan Province and Turkiye, the MS prevalence in this area is lower. Although MS prevalence and incidence in Azerbaijan are below European levels, they exceed those in some Asian countries.

**Conclusion:**

This is the first study on MS epidemiology in Southern Azerbaijan. The findings underscore the need for public awareness campaigns and enhanced diagnostic training for healthcare professionals, particularly in primary care, to facilitate early symptom detection and reduce diagnostic delays. Further research is recommended to investigate regional factors affecting MS diagnosis and to improve timely healthcare access.

## INTRODUCTION

Reliable national-level epidemiological studies are essential for developing effective healthcare policies for multiple sclerosis (MS), improving treatment measures, and raising awareness of its risk factors.^1^ Globally, MS prevalence increased by 20% between 2013 and 2023, with European countries experiencing a rise of up to 35%.^1^,^2^ In Switzerland, the prevalence of MS rose by approximately 20% between 2016 and 2021,^3^ while the prevalence among women aged 18–65 increased by 150–300% over the past three decades.^4^ According to the MS International Federation, over 80% of countries report rising MS prevalence.^1^ While MS was historically less prominent in Asia, recent decades have seen increasing prevalence across the region, including Arab countries.^5^,^6^ In neighboring Russia, higher MS prevalence is noted among Slavic populations compared to other ethnic groups.^7^ Epidemiological relevance is also highlighted in Turkiye and Iran.^8^-^12^ MS is also significant in Azerbaijan, as evidenced by several studies focusing on the clinical epidemiology of the disease.^13^,^14^ The aim of this study was to investigate clinico-epidemiological characteristics of MS in the southern region of Azerbaijan.

## METHODS

This is a prospective, single-center, longitudinal study involving patients referred from regional clinics or those who presented directly. Patients were followed over a 10 years period (from January 1, 2013 to December 31, 2022) to observe clinical and epidemiological outcomes. Examination was conducted by a special expert commission at the Neurology Center of the Ministry of Health as part of a State Program and followed the national “Clinical Protocol for the Diagnosis and Treatment of Multiple Sclerosis”.

### Ethical Approval:

This study was approved by the Ethics Committee of Neurology Centre of Ministry of Health of the Republic of Azerbaijan on December 24, 2012 under approval reference 11/2012, covering all aspects of the nationwide study titled “Clinico-Epidemiological study of Multiple Sclerosis in the Republic of Azerbaijan”. This manuscript presents findings specific to the Lankaran-Astara economic region within the scope of the approved protocol.

### Geographical and socio-demographic characteristics of the region:

The southern region of Azerbaijan includes the Lankaran-Astara economic region. This region borders the Caspian Sea to the east and Iran to the south and west. The economic region covers an area of 6.07 thousand km², accounting for 7.01% of the country’s territory, with a humid subtropical climate. It is densely populated, with a population density of 153 people per km², representing 9.19% of the country’s population, of which 50.47% are men. The majority of the population (73.63%) resides in rural areas.^15^

### Study Subjects.

During the study period, 123 individuals from the region visited the center; 104 were confirmed to have MS (new or reconfirmed diagnoses), while 19 had their diagnosis ruled out. Diagnosed patients were included in a digital database.

### Inclusion Criteria:

- Patients with a confirmed diagnosis of MS based on the national “Clinical Protocol for the Diagnosis and Treatment of Multiple Sclerosis.

### Exclusion Criteria:

- Patients with incomplete data, those unable to complete the necessary diagnostic evaluations, or those who received other diagnoses or were misdiagnosed were excluded from the study.

### Statistical analysis:

Incidence and prevalence rates per 100,000 people were calculated, with age-standardized rates determined using direct standardization methods. Reference populations included the WHO Standard (2000–2025), the European Standard (2011–2030), and Azerbaijan’s population age and sex distribution (for comparison across regions within the country) as of 01.012023.^15^ Statistical analysis was performed using IBM SPSS Statistics 27, applying Student’s t-test and the Mann-Whitney U test, with the null hypothesis being rejected at p<0.05.

## RESULTS

Among 104 MS patients, 46.2% were men (48) and 53.8% women (56), with 68.3% residing in rural areas. The mean age at diagnosis was 36.31±6.93 years, ranging from 16 to 47, with no significant differences by sex or residence (p>0.05), Table-I. The mean age at the first probable attack was 29.30±7.51 years, with no significant urban-rural differences (p>0.05). The diagnostic delay averaged 7.01±5.12 years. By the end of the study (December 31, 2022), two patients had died. Among the remaining 102 patients, disease duration was significantly longer in rural residents (14.39±6.93 years) compared to urban residents (p<0.05).

**Table-I:** Comparison of age and other indicators of multiple sclerosis patients by sex and place of residence.

Indicator		N	Min.	Max.	Median	Mean	SE	SD	P_U_
Age at Diagnosis	Male	48	16	47	36.50	35.77	1.02	7.09	0.489
	Female	56	18	47	37.00	36.77	0.91	6.82
	Urban	33	25	47	37.00	36.79	1.22	7.01	0.706
	Rural	71	16	47	37.00	36.08	0.82	6.93
	Total	104	16	47	37.00	36.31	0.68	6.93	
Age at Probable First Attack	Male	48	15	47	28.50	28.92	1.10	7.65	0.570
	Female	56	14	47	29.00	29.63	1.00	7.44
	Urban	33	18	47	31.00	31.12	1.31	7.55	0.097
	Rural	71	14	47	28.00	28.45	0.88	7.40
	Total	104	14	47	29.00	29.30	0.74	7.51	
Diagnostic delay	Male	48	0	20	6.00	6.85	0.68	4.67	0.961
	Female	56	0	23	6.50	7.14	0.74	5.51
	Urban	33	0	13	5.00	5.67	0.60	3.46	0.157
	Rural	71	0	23	7.00	7.63	0.67	5.64
	Total	104	0	23	6.00	7.01	0.50	5.12	
Duration of Disease	Male	47	2	27	11.00	12.70	0.92	6.28	0.360
	Female	55	3	29	12.00	14.00	0.88	6.54
	Urban	32	3	21	11.00	11.25	0.80	4.52	0.037
	Rural	70	2	29	14.00	14.39	0.83	6.93
	Total	102	2	29	12.00	13.40	0.64	6.42	

***Note:*** N – number of patients, SE – standard error of the mean, SD – standard deviation. Statistical significance of differences (comparison by sex and place of residence): P_U_ – by Mann-Whitney U test (abbreviations are consistent across all other tables).

The distribution of MS types at diagnosis highlights the predominance of Relapsing-Remitting MS (RRMS), accounting for 80.8% of cases (84 patients). This aligns with expectations, as RRMS is the most common clinical course observed in early MS diagnoses. Progressive forms were less prevalent: Secondary Progressive MS was identified in 15.4% of patients (16 persons), while the Progressive Relapsing MS (PRMS) form accounted for only 2.9% (three patients). Notably, no cases of Primary Progressive MS (PPMS) were recorded in this cohort, and Clinically Isolated Syndrome was rare, comprising just 1% of diagnoses. These findings emphasize the dominance of RRMS at initial presentation, with progressive forms emerging less frequently.

By 31.12.2022, the prevalence of MS in the Lankaran-Astara economic region was 10.96±1.09 per 100,000 populations, with rates of 10.01±1.46 in men and 11.93±1.61 in women, Table-II. Although prevalence was higher in women, the gender difference was not statistically significant (p>0.05). The 40–44 age group had the highest prevalence (34.73±7.24), followed by the 35–39 age group (29.17±6.22), with similar patterns observed for gender-specific rates (p>0.05).

**Table-II:** Age specific Prevalence of MS as of December 31, 2022.

Age Groups	Male		Female		Urban		Rural		Total	

	N	P±SE	N	P±SE	N	P±SE	N	P±SE	N	P±SE
<20	0	0	0	0	0	0	0	0	0	0
20-24	1	3.08±3.08	0	0	0	0	1	2.13±2.13	1	1.61±1.61
25-29	3	7.94±4.58	5	13.59±6.08	2	10.34±7.31	6	10.86±4.43	8	10.73±3.79
30-34	5	11.92±5.33	3	7.18±4.15	4	18.43±9.22	4	6.45±3.22	8	9.55±3.38
35-39	13	34.28±9.51	9	24.00±8.00	6	30.49±12.45	16	28.71±7.18	22	29.17±6.22
40-44	10	29.87±9.44	13	39.69±11.01	9	51.30±17.09	14	28.75±7.68	23	34.73±7.24
45-49	5	17.14±7.66	10	34.53±10.92	4	25.60±12.80	11	25.87±7.80	15	25.80±6.66
50-54	6	22.18±9.05	8	28.41±10.04	4	26.60±13.30	10	24.89±7.87	14	25.35±6.78
55-59	3	12.17±7.03	4	14.44±7.22	2	13.58±9.60	5	13.28±5.94	7	13.37±5.05
60-64	1	4.72±4.72	2	7.88±5.57	1	7.14±7.14	2	6.14±4.34	3	6.44±3.72
≥65	0	0	1	2.57±2.57	0	0	1	2.20±2.20	1	1.49±1.49
Total	47	10.01±1.46	55	11.93±1.61	32	13.04±2.30	70	10.22±1.22	102	10.96±1.09

***Note:*** P – prevalence rate per 100,000 people.

Urban prevalence (13.04±2.30 per 100,000) was higher than rural prevalence (10.22±1.22 per 100,000), but the difference was not statistically significant (p>0.05). In both urban and rural areas, the highest prevalence occurred in the 40–44 age group: 51.30±17.09 and 28.75±7.68 per 100,000, respectively, Table-II. Age-standardized prevalence, adjusted for the population of Azerbaijan, revealed no significant differences by gender or place of residence Table-III. Because of the strict quarantine measures implemented in response to the COVID-19 pandemic in 2020, admissions of MS patients to the Neurological Center were restricted, resulting in only two patients being diagnosed that year. Excluding 2020, the annual number of new MS diagnoses from 2013 to 2022 ranged from 8 to 11 patients.

**Table-III:** Age-Standardized Prevalence of Multiple Sclerosis (as of 31.12.2022).

Sex	P±SE (WSt)	P±SE (EuSt)	P±SE (AzSt)
Males	9.16±1.40	9.75±1.44	10.32±1.48
Females	10.73±1.53	12.06±1.62	11.89±1.61
Rural	9.38±1.17	10.44±1.23	10.43±1.23
Urban	11.64±2.18	12.51±2.26	13.13±2.31
All patients	9.97±1.04	10.96±1.09	11.14±1.09

***Note:*** WSt – World Standard, EuSt – European standard, AzSt – Azerbaijan Standard.

The average 10-years incidence of MS was 0.94±0.32 cases per 100,000 populations (Table-IV), with no significant differences between genders (p>0.05). Although the urban incidence was five times higher than the rural rate, the difference was not statistically significant (p>0.05). Age standardization using Azerbaijan’s population revealed no notable differences. In 2020, strict quarantine measures contributed to a low incidence rate. Excluding 2020, the standardized urban incidence (2.59±1.03 per 100,000) was significantly higher than the rural rate (0.49±0.27 per 100,000; p<0.05) Table-IV.

**Table-IV:** Multiple Sclerosis Incidence by Sex and Place of Residence.

Indicator		I±SE	I±SE (WSt)	I±SE (EuSt)	I±SE (AzSt)
Mean 2013-2022	Males	0.91±0.44	0.85±0.43	0.79±0.41	0.93±0.45
	Females	0.97±0.46	0.90±0.45	0.85±0.43	0.99±0.47
	Rural	0.45±0.26	0.41±0.25	0.39±0.24	0.44±0.26
	Urban	2.28±0.97	2.19±0.94	2.03±0.91	2.42±0.99
	All patients	0.94±0.32	0.87±0.31	0.82±0.30	0.96±0.32
Mean (2020 excluded)	Males	0.99±0.46	0.92±0.45	0.86±0.43	1.01±0.47
	Females	1.05±0.48	0.98±0.46	0.92±0.45	1.07±0.49
	Rural	0.50±0.27	0.46±0.26	0.44±0.26	0.49±0.27
	Urban	2.45±1.00	2.35±0.98	2.17±0.94	2.59±1.03*
	All patients	1.02±0.33	0.95±0.32	0.89±0.31	1.04±0.34

***Note:*** I – incidence rate per 100,000 people; Statistical Significance of Difference: P_t_ – by t-Student test (comparison by place of residence),

* – P<0,05.

## DISCUSSION

Numerous epidemiological studies on MS have been carried out in Azerbaijan.^13^,^14^ However, data on the incidence and prevalence of MS in the southern region have not been investigated until now. This study fills that gap and allows for comparison with similar studies conducted in other countries. Diagnostic delay was 7.01±0.5 years, significantly higher than Azerbaijan’s national average of 5.2±0.1 years (p<0.001).^14^ The higher diagnostic delay may be attributed to the shortage of physicians (8.9 per 10,000 population) and the limited capacity of outpatient clinics (69.7 per 10,000 population) in the region, compared to the national averages of 32.4 and 105.3 per 10,000 population, respectively.^16^ Diagnostic delays vary globally: in Iran, it was ~18 months (1989–2008);^11^ in Egypt, 18.63 months (2021–2023).^17^ In the U.S., delays were seven years in 2006, 4.2 years (1982–2001), and 1.1 years after 2017.^18^,^19^ In the Scottish Highlands, diagnostic delay averaged 38.8±43.2 months, while in Ireland, it ranged from 2.5 to 4.2 years, depending on MS clinical course.^20^,^21^ A higher level of public health awareness and diagnostic capabilities may be a reason of shorter diagnostic delay in these countries.

The prevalence of MS in the Lankaran-Astara economic region was 10.96±1.09 per 100,000 people, which is lower than the corresponding figure in the East Azerbaijan Province of Iran (27.7 per 100,000 people), a region bordering Azerbaijan with a predominantly ethnic Azerbaijani population.^12^ In Turkiye, in the districts of Geyve and Kandira the prevalence of MS among the rural population was 58.5 and 33.1 cases per 100,000 population, respectively.^8^ In the highly urbanized city of Sivas recorded an MS prevalence of 288 cases per 100,000 populations, marking a peak rate for Turkiye.^9^ Thus, the prevalence of MS in the regions of Türkiye was significantly higher than in Azerbaijan. The lower prevalence rate of MS in our findings compared to Türkiye may be attributed to their use of a community-based, door-to-door survey methodology, which likely identified undiagnosed cases that our center-based approach might have missed. Additionally, a nationwide epidemiologic overview in Türkiye reported a prevalence of 96.4 per 100,000 population, which is also notably higher.^10^

The prevalence of MS among ethnic groups in the North Caucasus region of Russia (8.0–15.2 per 100,000 people) is comparable to our findings but lower than that among Russians (45.0 per 100,000).^7^ However, the prevalence and incidence reported in the multiethnic Republic of Dagestan (5.7 and 0.2 per 100,000, respectively), which borders Azerbaijan, are lower than those in our study. The similarity between the prevalence among ethnic groups in the North Caucasus and our findings, as well as the lower rates compared to Russians, may be explained by epigenetic factors and lifestyle differences.^7^ The lower rates observed in Dagestan may result from delayed healthcare-seeking behavior or limited access to diagnostic facilities.

The epidemiological indicators of MS in Azerbaijan remain low compared to those in the United States and European countries, but they are higher than the results of many studies conducted in Asian countries.^1^,^2^,^4^,^18^-^24^ Prevalence of MS in Pakistan estimated to be similar (10 patients per 100000 population) to our findings.^25^,^26^ Nevertheless, in some studies, the prevalence of MS in Asian countries was found to be higher than in the southern region of Azerbaijan. Specifically, in northern Japan, the prevalence was 18.6 cases per 100,000 people.^24^ In Irbid and Amman (Jordan), the prevalence of MS reached 38 and 39 cases per 100,000, respectively.^27^ In Israel, the age-standardized prevalence was 61.6 cases per 100,000 among Jews and 35.3 cases per 100,000 among other nationalities.^28^ A higher level of public health awareness and better diagnostic capabilities in these Asian countries could lead to more accurate prevalence estimates.^29^

This study provides the first epidemiological data on multiple sclerosis (MS) in the southern region of Azerbaijan, highlighting a significant diagnostic delay and a lower prevalence compared to neighboring and other regions. Its key strength lies in addressing a previously unexplored area, offering valuable insights into the regional disparities in MS prevalence and diagnostic timelines. These findings underscore the need for targeted public health interventions, such as improving healthcare infrastructure and awareness, while future studies should explore the impact of genetic, environmental, and healthcare accessibility factors on MS epidemiology in this region

### Limitations:

The study population did not include individuals who were not diagnosed with MS at the primary care level, those who received other diagnoses or were misdiagnosed, and individuals with mild symptoms who never sought medical attention or visited a healthcare center.

## CONCLUSION

The results obtained show that in the southern region of Azerbaijan, diagnostic delay significantly exceeds the countrywide average and is generally higher compared to other countries. This indicates existing difficulties in the early diagnosis of the disease in the region. On one hand, efforts are needed to raise public awareness about the symptoms of MS. On the other hand, it is crucial to improve the attention of healthcare professionals, particularly in primary care, to the issues of differential diagnosis of MS. This may involve educational programs for physicians aimed at improving the detection of early symptoms of the disease and timely referral of patients to tertiary centers. Improving MS diagnosis requires closer collaboration between primary and specialized care to accelerate detection and enhance treatment quality.

### Recommendations:

Further research is needed to identify factors delaying diagnosis, including barriers related to healthcare access, patient awareness, or regional disease characteristics. Such studies will help address obstacles to timely consultation and improve diagnostic efficiency.

### Author’s Contribution:

**RRA:** Conceived, designed and did statistical analysis & editing of manuscript, is responsible for integrity of research.

**SNM:** Helped in designing, did data collection and manuscript writing.

**RKS:** Supervised the entire study, helped in data interpretation, did review.

All authors have approved the final version and are accountable for the integrity of the study.
